# Breast Cancer and Meningioma Risk: A Mendelian Randomization Study

**DOI:** 10.1002/brb3.70422

**Published:** 2025-03-14

**Authors:** Jian‐Wei Huang, Yi‐Fei Wang, Ying‐Qing Hu, Hai‐Yong He, Shuang‐Qi Gao, Ying Guo

**Affiliations:** ^1^ Departments of Neurosurgery, The Third Affiliated Hospital Sun Yat‐Sen University Guangzhou Guangdong Province China; ^2^ Department of Medical Ultrasound, The Third Affiliated Hospital Sun Yat‐Sen University Guangzhou Guangdong Province China; ^3^ Department of Anesthesiology, The Fifth Affiliated Hospital Sun Yat‐Sen University Zhuhai Guangdong Province China

**Keywords:** Mendelian randomization, breast cancer, meningioma

## Abstract

**Background:**

The exact nature of the link between breast cancer and meningiomas is unknown, although observational studies have shown a correlation between the two. Using a two‐sample Mendelian randomization (MR) strategy, we aimed to investigate the effect of breast cancer on meningiomas.

**Methods:**

Three sets of genetic instruments were utilized in this study based on publicly available genetic summary data. For breast cancer, we selected 62 strongly associated SNPs; separate datasets were curated for HER2‐positive and HER2‐negative subtypes. MR analyses included outlier testing, MR‐Egger regression, MR‐PRESSO, weighted median, and inverse variance weighted approaches.

**Results:**

The inverse variance weighted analysis demonstrated significant evidence for breast cancer's effect on meningioma risk (OR = 1.213, 95% CI = 1.054–1.396, *p* = 0.007), supported by MR‐Egger (OR = 1.456, 95% CI = 1.066–1.988, *p* = 0.021) though not by the weighted median method (OR = 1.095, 95% CI = 0.914–1.311, *p* = 0.326). Inverse variance weighting specifically revealed a significant association between HER2‐positive breast cancer and meningioma incidence (OR = 1.203, 95% CI = 1.048–1.381, *p* = 0.009). Furthermore, breast cancer risk was associated with an increased incidence of malignant meningiomas (OR = 1.64, 95% CI = 1.12–2.40, *p* = 0.011).

**Conclusions:**

This represents the first MR study investigating the causal relationship between breast cancer and meningiomas. Our findings support the hypothesis that breast cancer may increase meningioma risk.

## Graphical abstract

This is a large‐scale GWAS and MR study linking breast cancer to an increased risk of meningiomas and finding that breast cancer, especially HER‐2 positive breast cancer, raises the risk of meningiomas, which suggests genetic causation and has important clinical implications for the prevention and treatment of meningiomas.

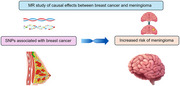

## 1 Background

The most common type of brain tumor is a meningioma, which accounts for over one‐third of all primary tumors affecting the central nervous system (CNS) (Ogasawara et al. 2021). However, compared to gliomas, our understanding of the epidemiology of meningiomas, including the statistics on incidence and the examination of risk factors, remains relatively limited (Baldi et al. 2018). According to previous research, key risk factors for the disorder include being overweight, a history of exposure to ionizing radiation, and a history of allergic reactions. Previous observational studies have identified correlations between the risk of meningioma in older women and factors such as physical activity, body mass index (BMI), height, a history of breast cancer, and uterine fibroids (Johnson et al. 2011). To further understand meningiomas and develop strategies to reduce their incidence, it is crucial to focus on other potentially modifiable risk factors.

Over the course of several decades, there has been speculation about the possible connection between meningiomas and breast cancer (Lieu et al. 2003; Salvati and Cervoni 1996; Burns et al. 1986). However, as of now, there is no conclusive evidence on the topic. A total of 51 retrospective papers were identified, providing information on 2238 patients who suffered from both conditions. These studies included case reports, case series, and cancer registry reports. We were able to conduct a prevalence analysis and meta‐analysis on 18 of these studies. According to a meta‐analysis of 13 studies using random effects, female meningioma patients had a significantly higher prevalence of breast cancer compared to the general population (odds ratio [OR], 9.87; 95% confidence interval [CI], 7.31–13.32). This difference became evident when comparing these patients to the overall population. Although breast cancer patients (11 studies) showed an increased risk of meningioma compared to the baseline population, the random‐effects model found no statistically significant difference (OR, 1.41; 95% confidence interval, 0.99–2.02) (Degeneffe et al. 2023). There are weak or nonexistent associations between initial breast cancer and subsequent meningioma, and between initial meningioma and subsequent breast cancer in female patients, according to previous cohort studies (Goh et al. 2024, Criscitiello et al. 2014; Bonito et al. 1993). When genders were differentiated, it became clear that meningiomas are strongly linked to breast cancer in women. However, in men, a clear association was not observed due to (Rao et al. 2009). Upon further examination, it was found that the probability of meningiomas was significantly increased in women with aggressive breast cancer, with the probability being 26 percent (Lopez‐Rivera et al. 2020).

In breast cancer, the HER‐2/neu oncogene, which belongs to the erbB‐like oncogene family, is related to the epidermal growth factor receptor but remains distinct from it. It has been demonstrated that human breast cancer cell lines exhibit increased expression of this gene (Slamon et al. 1987). Emerging evidence indicates that the proto‐oncogene Her‐2/neu, or c‐erb‐B2, plays a significant role in breast cancer prognosis and prediction. Patients with breast cancer who overexpress or amplify Her‐2/neu tend to have a poorer prognosis (Kaptain et al. 2001). In the field of breast cancer, which is rapidly expanding in both diagnostic and therapeutic domains related to biological progression, receptors within the HER family play a crucial role. The dimerization of HER‐2 with other HER family members, such as HER‐3, serves as a key driver for the proliferation and survival of tumor cells. According to numerous studies, overexpression of the HER‐3 gene is linked to a worse prognosis (Stanek et al. 2022).

These biases can be circumvented through Mendelian randomization (MR), which utilizes genetic polymorphisms as exposure indices to establish causality between risk factors and disease (Chen et al. 2020, 2021, 2020, 2021). If an exposure (e.g., breast cancer) causally influences an outcome (e.g., meningioma), genetic variations affecting breast cancer would likely also impact meningioma to some extent. However, horizontal pleiotropy must first be excluded—an independent mechanism whereby genetic variations affect meningioma through distinct biological pathways (Ding et al. 2023; Zhong et al. 2023).

We utilized summary statistics from a large genome‐wide association study (GWAS) on meningiomas, HER2‐positive breast cancer, HER2‐negative breast cancer, and overall breast cancer to conduct a two‐sample MR analysis in this investigation. Out of 20,126 breast cancer patients and 1605 meningioma patients, 12,081 and 1573, respectively, were included in the GWAS. The primary motivation behind this research was to determine whether breast cancer increases the risk of meningioma. Our findings suggest that breast cancer plays a causal role in the risk of developing meningioma.

## 2 Methods

### 2.1 Data Sources and Participants in the Study

This analysis made use of data obtained from the European Sample FinnGen project (https://www.finngen.fi/en). The data that were utilized in this investigation included information on breast cancer and meningioma. This European sample is a database that is open‐source and open, and it has been subjected to stringent quality verification. In its capacity as the body responsible for monitoring the FinnGen project, the University of Helsinki has made certain that every study contained within the dataset has been granted authorization by the local institutional review board and ethics committee. As a result, there is no longer a requirement for providing additional ethical approval (Yuan et al. 2023; Kurki et al. 2023).

### 2.2 Statistical Analyses

**FIGURE 1:**
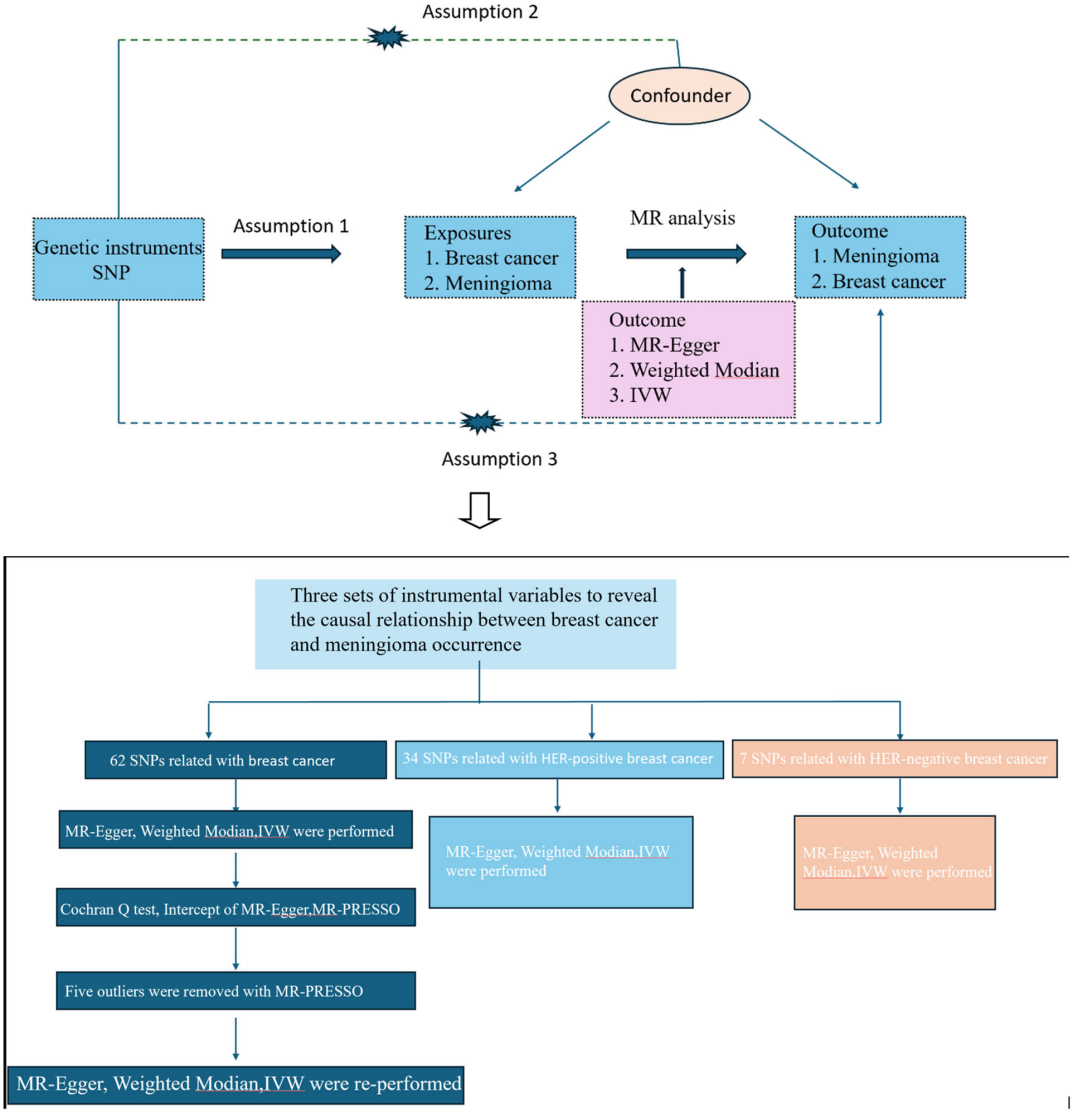
Workflow of Mendelian randomization study revealing causality from depression on prostate carcinogenesis. IVW, inverse variance weighted; MR, Mendelian randomization; MR‐PRESSO, MR pleiotropy residual sum and outlier; SNP, single‐nucleotide polymorphism.

MRCANO (1.0) and the TwoSampleMR (0.5.6) package were utilized in the process of doing the data analysis for this work, which was carried out with R (version 4.2.1). In order to find out what causes complex disorders, the MR method uses genetic variants' single‐nucleotide polymorphisms (SNPs) as instrumental variables (IVs) (Pan et al. 2023; Wang et al. 2023). Once the GWAS effector alleles for breast cancer and meningioma were reconciled, we utilized a number of different MR methods in order to determine the MR estimates for both conditions (Figure 1). Specifically, we utilized IVW, weighted median, MR‐Egger, and weighted median. Due to the fact that each of the underlying assumptions was different, a variety of strategies were utilized (J. Li, Tang et al. 2023).

The major result was determined by doing an inverse variance weighted (IVW) meta‐analysis of Wald ratios for individual SNPs. This was done under the assumption that the tool affects the outcomes purely through the exposure of interest, without any other pathways being considered. For the purpose of supplementing the IVW estimates, the MR‐Egger and weighted median methods were utilized. These approaches produce estimates that are more reliable across a wide range of scenarios, despite the fact that they are less efficient (they produce wider confidence intervals overall) (Dai et al. 2023; Yu et al. 2024).

When there is a possibility that heterogeneity in MR estimates may be severely impaired, sensitivity analyses are absolutely necessary for determining whether or not there is a possibility of pleiotropy in MR research. Hence, we used a heterogeneity marker that was created using the IVW technique (Cochran Q‐derived *p* < 0.05) to find out if horizontal pleiotropy was possible. The intercept that was obtained from MR‐Egger regression was used as a marker for directional pleiotropy, and the presence of this phenomenon was shown by a *p* value that was less than 0.05 throughout the analysis. For that reason, this study used the MR‐Egger regression in addition to the main IVW analytic approach to conduct the heterogeneity test. This investigation did not reveal any evidence of heterogeneity among these IVs, since the *p* value was more than 0.05 (Zhang et al. 2024; Ji and Shu 2023).

First, MR‐PRESSO finds horizontal polytomies; second, it uses outlier elimination to fix horizontal polytomies; and third, it compares causal estimates before and after using outlier correction to see if there are significant differences. Horizontal polytropy shows less bias and better accuracy when the variance % is less than 10%, according to IVW and MR‐Egger. This stands in contrast to the other two approaches (M. Li, Jiang, et al. 2023; Z. Li, Jin, et al. 2023; O'Hagan et al. 2023). In this work, the *p* value obtained from the pleiotropy test was utilized to conduct an analysis of the presence of pleiotropy. The probability of polytropy in the causal analysis was considered to be minor or nonexistent if the *p* value was greater than 0.05. When this occurred, the influence of polytropy may be disregarded. In order to determine whether or not the MR estimations were influenced or biased by a single SNP, a leave‐one‐out analysis was carried out (Shen et al. 2024; Z. Li et al. 2024).

## 3 Results

**FIGURE 2:**
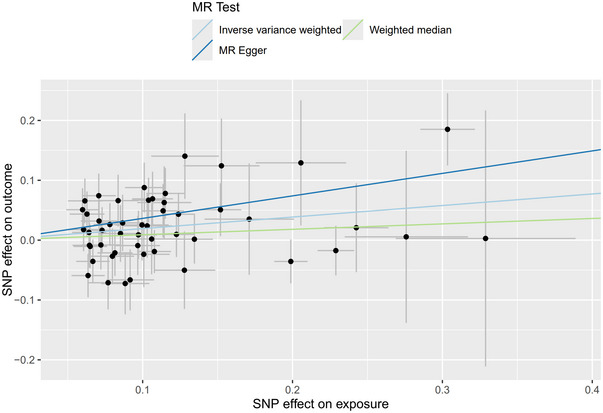
Scatter plot of genetic association between breast cancer and meningioma.

In the bidirectional MR analysis, 62 SNPs were extracted with breast cancer as the exposure and meningioma as the outcome. The results demonstrated that IVW (OR = 1.213, 95% CI = 1.054–1.396, *p* = 0.007), MR‐Egger (OR = 1.456, 95% CI = 1.066–1.988, *p* = 0.021), and weighted median (OR = 1.095, 95% CI = 0.914–1.311, *p* = 0.326) indicated that breast cancer had a causal effect on meningioma (Table 1 and Figure 2). Additionally, the results for HER‐positive breast cancer as the exposure and meningioma as the outcome showed that IVW (OR = 1.203, 95% CI = 1.048–1.381, *p* = 0.009) suggested HER‐positive breast cancer also had a causal effect on meningioma (Table 1). However, the *p* values in the remaining results were greater than 0.05, indicating no causal relationship between meningioma and breast cancer.

**TABLE 1:** Mendelian randomization estimates of the relationship between breast cancer and meningiomas.

Exposure	Outcome	Method	Beta	SE	OR	OR1	OR2	Pval
Breast cancer	Meningioma	IVW	0.19291	0.07167031	1.21277	1.05383	1.39568	0.00711
		MR‐Egger	0.37584	0.15904882	1.45622	1.06621	1.9889	0.02182
		Weighted median	0.09049	0.09212509	1.09471	0.91386	1.31135	0.32597
HER‐positive breast cancer	Meningioma	IVW	0.1848	0.07050744	1.203	1.0477	1.3812	0.0088
		MR‐Egger	0.2885	0.15924625	1.3344	0.9767	1.8233	0.0794
		Weighted median	0.0815	0.08538911	1.0849	0.9177	1.2826	0.3397
ER‐negative breast cancer	Meningioma	IVW	0.2754	0.1953329	1.3171	0.8982	1.9315	0.1585
		MR‐Egger	1.1437	0.4350992	3.1384	1.3377	7.3633	0.0466
		Weighted median	0.039	0.1602496	1.0398	0.7595	1.4235	0.8077
Meningioma	Breast cancer	IVW	0.0814	0.06073135	1.0848	0.9631	1.2219	0.1802
		MR‐Egger	−0.172	0.09538609	0.8416	0.6981	1.0146	0.3217
		Weighted median	0.0344	0.03922292	1.035	0.9585	1.1178	0.3798

Subsequent heterogeneity analysis revealed significant heterogeneity in the results of the IVW method for breast cancer versus meningioma (Q‐pval = 0.000377), which was much less than 0.05, indicating the presence of significant heterogeneity in the results. To address this issue, we conducted an additional MR‐PRESSO analysis on the results and identified five outliers (rs12253527, rs1292010, rs62235753, rs68056147, and rs9871261). After removing these five outliers, we reanalyzed the heterogeneity of the resulting data and found that *p* = 0.256 > 0.05, indicating no heterogeneity among these IVs (Table 2).

**TABLE 2:** Heterogeneity of IVW and MR‐Egger tests for directional pleiotropy.

Heterogeneity					
Exposure	Outcome	Method	*Q*	*Q*_df	*Q*_pval
Breast cancer	Meningioma	MR Egger	93.12234	53	0.000551
		IVW	96.03019	54	0.000377
HER‐positive breast cancer	Meningioma	MR Egger	58.34264	32	0.002999
		IVW	59.30797	33	0.0033
ER‐negative breast cancer	Meningioma	MR Egger	10.98801	5	0.051618
		IVW	21.00711	6	0.001829
Meningioma	Breast cancer	MR Egger	0.450882	1	0.501916
		IVW	8.298938	2	0.015773
Heterogeneity after MR‐PRESSO					
Breast cancer	Meningioma	MR Egger	55.06023	48	0.225029
		IVW	55.06677	49	0.255922

**FIGURE 3:**
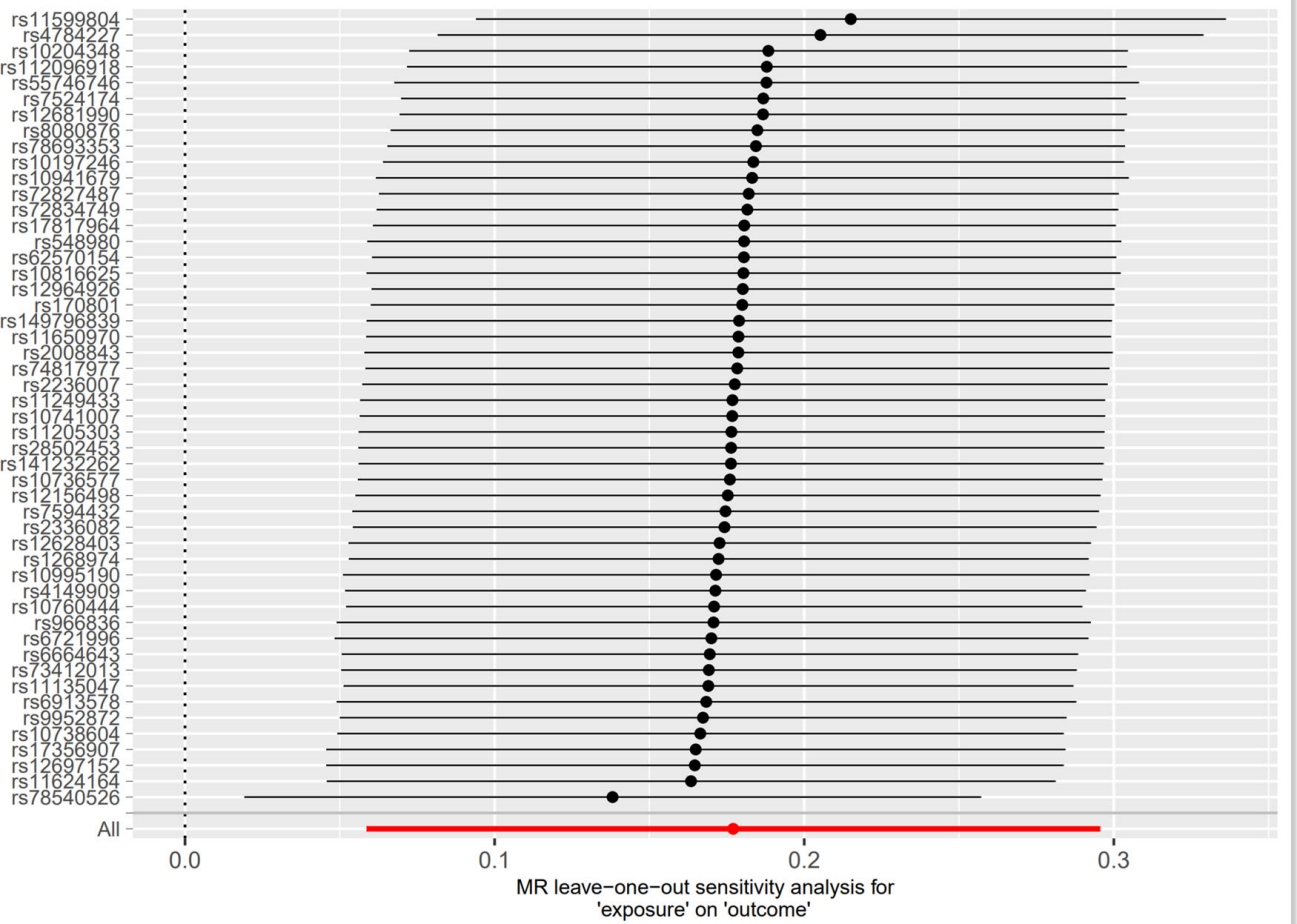
Causality map of breast cancer–related factors: breast cancer–related single‐nucleotide polymorphisms (SNPs) on meningiomas.

In the end, the data were examined to determine whether they possessed multivariate validity. The obtained *p* values were greater than 0.05, indicating that the results did not exhibit horizontal multivariate validity and that the results were stable and dependable (Table 3). The results of the leave‐one‐out analysis revealed no high‐impact areas, and the findings also showed no substantial anomalies (Figure 3). We found evidence supporting the hypothesis of a causal connection between breast cancer and meningioma, as well as a causal connection between HER2‐positive breast cancer and meningioma. However, the data do not support the hypothesis that meningioma is a causative factor in the development of breast cancer.

**TABLE 3:** Pleiotropy test of the relationship between breast cancer and meningiomas.

Pleiotropy test			
Exposure	Outcome	SE	*p*
Breast cancer	Meningioma	0.01761	0.20387
HER‐positive breast cancer	Meningioma	0.0226	0.47212
HER‐negative breast cancer	Meningioma	0.07649	0.08584
Meningioma	Breast cancer	0.0328	0.21827

**FIGURE 4:**
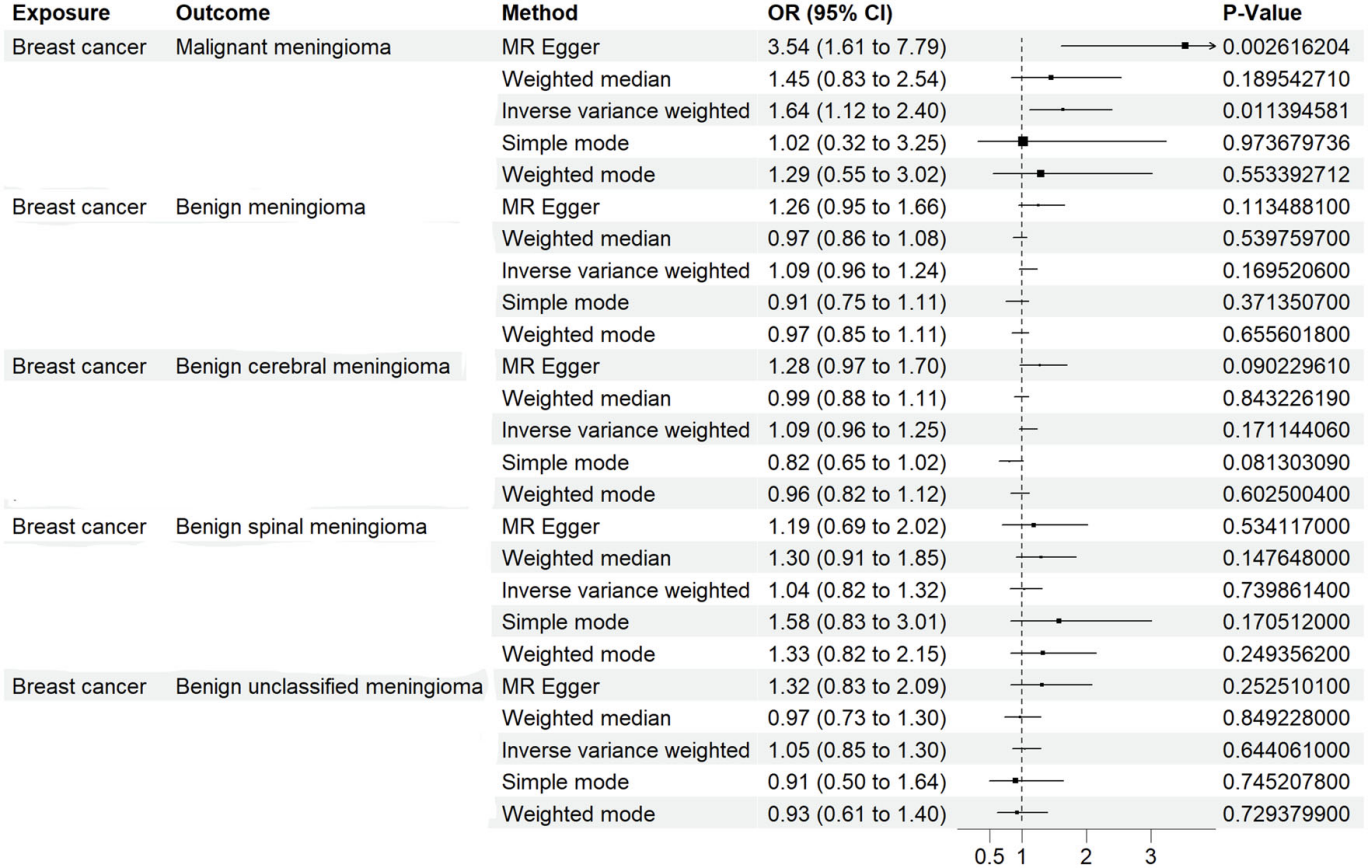
Scatterplot, funnel plot, and relationship between breast cancer and malignant meningioma. (a) Scatterplot of the instrumental association between breast cancer (*y*‐axis) and malignant meningioma (*x*‐axis) versus the slope of MR estimation. (b) Funnel plot of the instrumental accuracy (*y*‐axis) and Mendelian randomized association (*x*‐axis) between breast cancer and malignant meningioma. (c) Forest plot of the relationship between breast cancer and malignant meningioma obtained by leave‐behind medical analysis.

**FIGURE 5:**
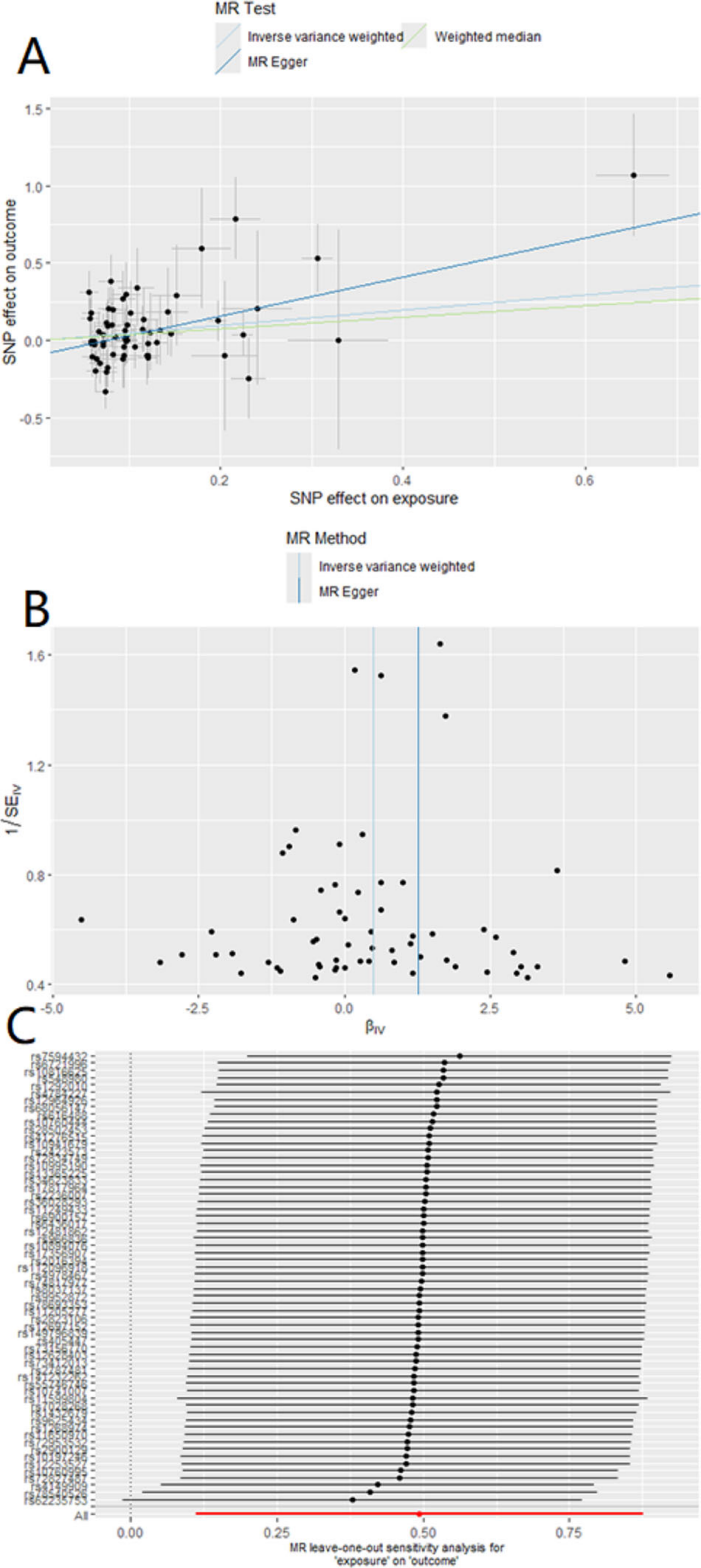
Mendelian randomization analysis of the correlation between breast cancer and the risk of various types of meningiomas. nsnp, number of single nucleotide polymorphisms; OR, ratio of ratios; CI, confidence interval; MR, Mendelian randomization.

To further validate our results from different perspectives, we incorporated a more detailed classification of meningiomas in subsequent analyses, including malignant meningioma, benign meningioma, benign central meningioma, benign chordoma, and benign unclassified meningioma (Yu et al. 2023). A two‐sample MR analysis, using breast cancer as the exposure factor and these meningioma subtypes as the outcomes, revealed that breast cancer genetically increases the incidence of malignant meningiomas (OR = 1.64, 95% CI = 1.12–2.40, *p* = 0.011). These findings were visualized using scatterplots, funnel plots, and forest plots (Figure 4). Additionally, we present the results for the other meningioma classifications simultaneously (Figure 5).

## 4 Discussion

We employed a two‐sample MR approach to comprehensively assess the causal relationship between breast cancer and the incidence of meningioma, uncovering substantial evidence that genetic predisposition to breast cancer affects the risk of meningioma.

Small‐ and medium‐sized effects were identified and examined through etiological analysis in MR, with data statistically analyzed using various MR methodologies. Our results contradict certain prior studies (Lieu et al. 2003; Jacobs et al. 1992). Nevertheless, the majority of prior research was observational in nature, failing to elucidate causality, suffering from chronological ambiguity, and lacking sufficient data to mitigate bias. Observational studies have been unable to establish a causal link between breast cancer and meningioma. Furthermore, all prior observational studies encountered challenges in addressing confounding factors and risk factor violations; by contrast, our application of the MR approach has allowed us to firmly establish causation while minimizing bias through an improved study design.

Our data indicate a causal link between breast cancer and meningioma incidence; however, the underlying physiological mechanisms remain unexplored. Numerous potential connections between breast cancer and meningiomas are broadly recognized at both molecular and behavioral levels. Previous studies indicate that the development of breast cancer is linked to endogenous estrogen. Meningiomas possess a significant quantity of estrogen and progesterone receptors, and the interaction between these steroid hormones and their specific receptors stimulates protein synthesis in the CNS, thereby facilitating the development of meningiomas (Poisson et al. 1984; Prossnitz and Barton 2023). This study establishes a causal association between breast cancer and meningioma by MR analysis. We hypothesize that breast cancer may impact the formation of meningiomas by altering the secretion of estrogen and progesterone. This study establishes a causal association between breast cancer and meningioma through MR analysis. We hypothesize that breast cancer may impact the formation of meningiomas by altering the secretion of estrogen and progesterone. The research we have conducted offers several benefits. First, our study is able to simulate a randomized controlled trial within an observational framework by utilizing a MR approach. Although widely accepted in causality research, randomized controlled trials are typically expensive and challenging to conduct systematically (Zhang 2023). MR also mitigates reverse causality issues compared to previous observational studies. The elucidation of the causative link between breast cancer and meningiomas holds significant implications for clinical practice, particularly in the management of metastases and meningiomas in breast cancer patients exhibiting neurological symptoms.

Nevertheless, there are some limitations to consider. From the beginning, every single GWAS dataset was derived from European populations. To ensure that our findings are consistent across various populations, additional research is required. Furthermore, it is of the utmost importance to acknowledge the differences among breast cancer patients. There is a possibility that certain stages of breast cancer are causally linked to meningiomas. The possibility of conducting a more in‐depth study that takes into account the myriad subtypes of breast cancer in the future should be considered.

## 5 Conclusion

This is the first MR study investigating the causal relationship between breast cancer and meningioma. Our MR study provides evidence supporting the hypothesis that breast cancer may increase the risk of meningioma.

## Author Contributions

**Jian‐Wei Huang**: writing–original draft, software, data curation. **Yi‐Fei Wang**: visualization, methodology. **Ying‐Qing Hu**: software, visualization. **Hai‐Yong He**: writing–review and editing, resources. **Shuang‐Qi Gao**: writing–review and editing, supervision, funding acquisition. **Ying Guo**: project administration, writing–review and editing.

## Conflicts of Interest

The authors declare no conflicts of interest.

### Peer Review

The peer review history for this article is available at https://publons.com/publon/10.1002/brb3.70422.

## Data Availability

All data used in the current study are publicly available GWAS summary data.
